# Supplementary material for: Alterations in Essential Fatty Acids, Immunoglobulins (IgA, IgG, and IgM), and Enteric Methane Emission in Primiparous Sows Fed Hemp Seed Oil and Their Offspring Response
Source: Vet Sci. 2022 Jul 11;9(7):352. doi: 10.3390/vetsci9070352 (PMC9319154; doi:10.3390/vetsci9070352)
Supplement: Supplementary file 1 [file vetsci-09-00352-s001.zip › vetsci-1802338-supplementary.pdf]

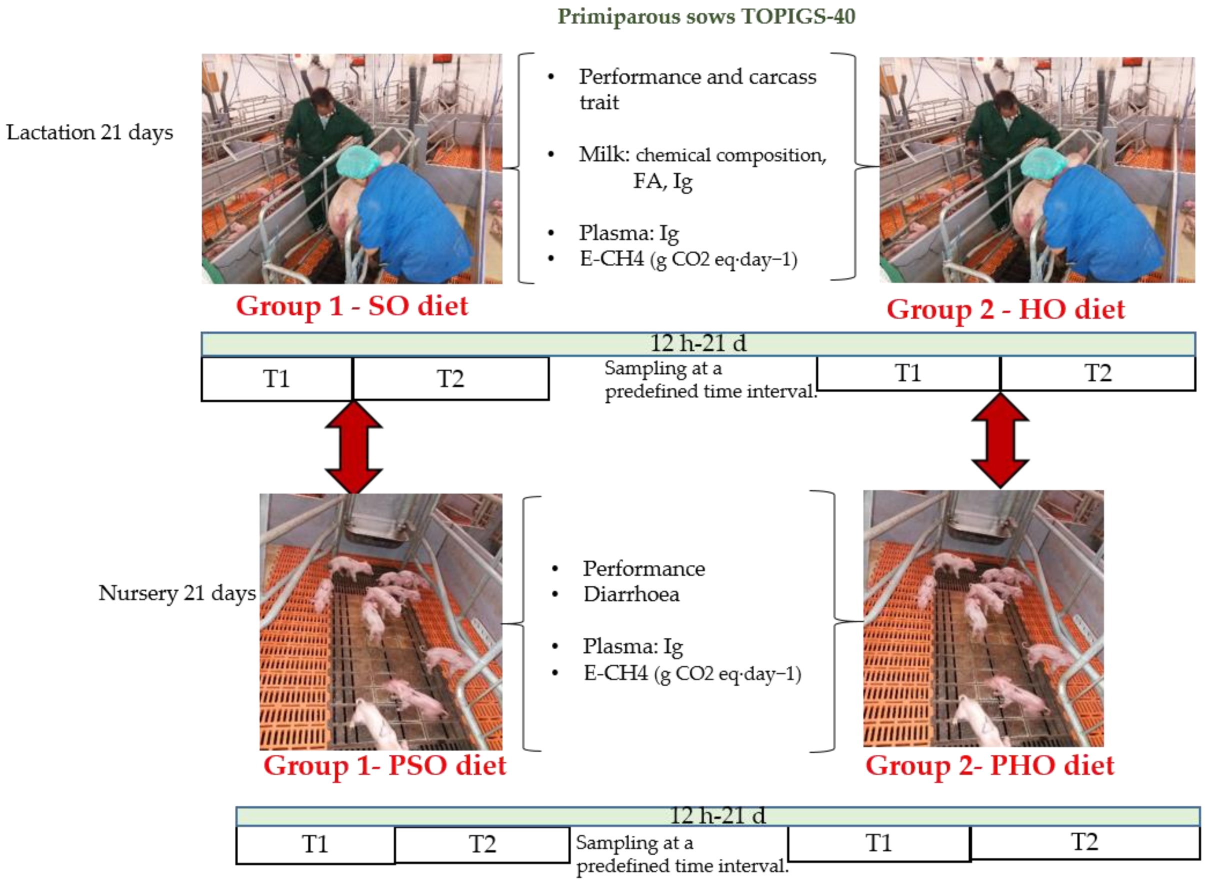

Figure S1. Experimental design

Table S1. Mean and SEM of sows feed intake, fat, dry matter, metabolizable energy

Items	Sows daily intake (kg x day^{-1})			
	**ADFI**	**FAT**	**DM**	**ME** (MJ/day)
Diet				
SO	4.84[a]	218.8[a]	4.25[a]	63.66[a]
HO	4.86[a]	219.3[a]	4.27[a]	63.90[a]
Period				
12 h-10 d	3.56[a]	0.161[a]	3.13[a]	46.9[a]
10 d-21 d	5.01[b]	0.227[b]	4.41[b]	66.0[b]
12 h-21 d	5.96[c]	0.269[c]	5.24[c]	78.4[c]
SEM	0.21	10.5	0.21	3.06
p-Value				
Diet	NS	NS	NS	NS
Period	**	***	***	***
Diet x Period	NS	NS	NS	NS

Abbreviations: average daily feed intake, ADFI; standard error of the mean, SEM.
[a, b, c] Different superscript letters indicate significantly different means; NS: nonsignificant effect; ** $p < 0.01$ and *** $p \leq 0.0001$ highly significant difference between means.

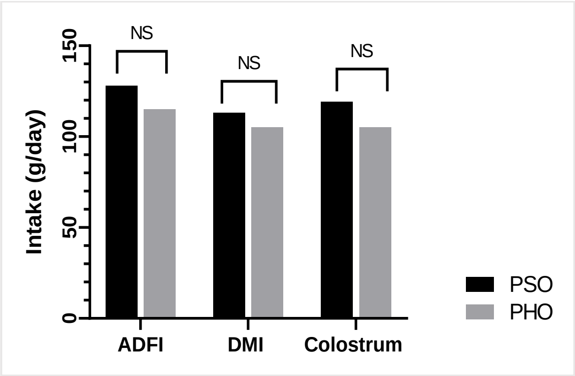

Figure S2. ADFI, fat and colostrum intake of piglets from sows fed a SO diet (PSO) or a HO diet based on a high level of n-3 fatty acids and lower n-6: n-3 ratio (PHO).

Abbreviations: average daily feed intake, ADFI; NS: nonsignificant effect ** $p < 0.01$ and *** $p \leq 0.001$ highly significant difference between means

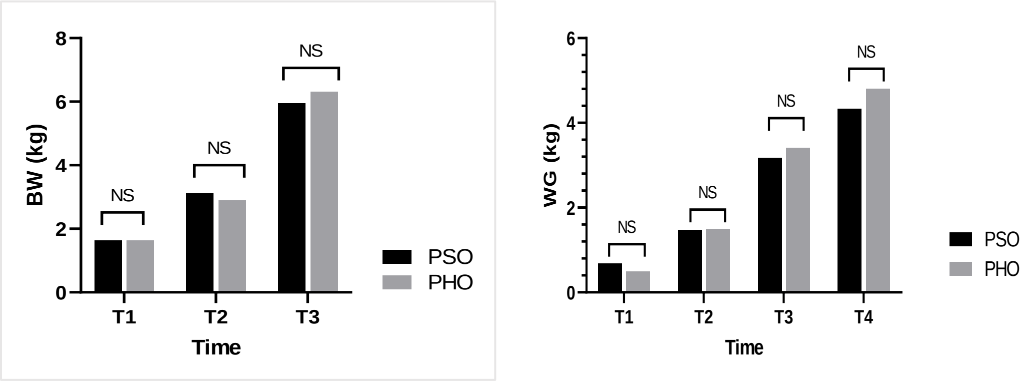

Figure S3. Growth parameters of piglets: BW of piglets from sows fed a SO diet (PSO) or a HO diet based on a high level of n-3 fatty acids and lower n-6: n-3 ratio (PHO) at different time points after farrowing: T1 (12h), T2 (10 days), and T3 (21days). Abbreviations: body weight, BW; PSO – piglets from sows fed SO diet; PHO – piglets from sows fed FO diet.
NS: nonsignificant effect.
